# Prevalence of mental health issues among teachers in China: the role of cultural values and psychological resilience

**DOI:** 10.3389/fpsyg.2026.1943721

**Published:** 2026-10-01

**Authors:** Chuan Ma

**Affiliations:** School of Marxism, East China University of Political Science and Law, Shanghai, China

**Keywords:** collectivistic values, cultural interventions, indigenous cultural resources, Mianzi (face concern), psychological distress, psychological resilience, teacher mental health, Zhongyong coping

## Abstract

**Introduction:**

Teachers worldwide experience occupational stress that threatens psychological wellbeing and educational effectiveness. Chinese teachers face substantial demands requiring culturally grounded approaches to psychological distress. Indigenous cultural resources may potentially buffer teachers against occupational adversity effectively.

**Methods:**

A cross-sectional survey recruited 750 teachers from three Chinese cities. Participants represented primary, secondary, and tertiary educational institutions across Jiangsu. Validated measures assessed workload, conflict, support, cultural values, coping, resilience, Mianzi. Psychological distress was measured using DASS-21 and K10 validated instruments. Reliability, descriptive, correlation, mediation, moderation, bootstrapping, and structural equation analyses followed. Sequential mediation and moderated mediation were examined using PROCESS Model 7.

**Results:**

Psychological distress prevalence was substantially high among participating Chinese teachers overall. Moderate-to-extremely severe depression affected 73.4% of teachers in this study. Moderate-to-extremely severe anxiety affected 80.5% of teachers in this study. Very high psychological distress affected 52.5% of teachers on K10. Workload and work-family conflict significantly increased psychological distress among teachers. Organizational support, collectivistic values, Zhongyong coping, and resilience provided protection. Collectivistic values indirectly reduced distress through sequential coping and resilience. Mianzi significantly strengthened this protective indirect pathway among affected teachers.

**Discussion:**

Findings demonstrate important protective roles for indigenous Chinese cultural resources. Zhongyong coping and resilience can strengthen psychological adaptation among teachers. Higher Mianzi amplified protective effects within the sequential mediation pathway. Culturally congruent interventions should combine institutional support with traditional psychological strengths. Such interventions may enhance teacher wellbeing within non-Western educational contexts.

## 1 Introduction

### 1.1 Research background and problem statement

The teaching profession has been known as one of the most emotionally draining jobs, as educators are required to constantly adapt to new expectations in education, engage in cognitive thinking, and manage their emotions and conversations with pupils (Savina et al., 2025; Bardach et al., 2022). In addition to the teaching job, teachers have been required to handle administrative, curriculum development, student behavioral issues, parental demands, and institutional accountability, yet demonstrate good professional performance (Nwoko et al., 2024). Teachers frequently experience chronic professional stress as a result of their various responsibilities, which negatively impacts their mental health and eventually lowers the caliber of instruction (Agyapong et al., 2022; Ghasemi, 2025). This has led to an international spotlight on the factors that impact psychological distress and mechanisms that safeguard the psychological wellbeing of teachers. Ensuring mental health among teachers has now become a crucial aspect of sustaining their career health and happiness in the rapidly changing education system, which has been as vital as it has been for the effective teaching and learning of students and for the overall social development (Rahimi and Oh, 2024; Long et al., 2023).

In the past 20 years, China has seen significant growth and reform in its education system, with growing demands for improved teaching standards, technological integration, curriculum innovation, and learning outcomes (Luo, 2023; Zhuang and Liu, 2022). At the same time, the teacher has been expected to attend to the challenge of the profession whilst coping with family duties, which has been likely to cause conflict between the two roles: the role of the teacher and the role of the parent (Junça-Silva and Freire, 2022).

Excess responsibilities, insufficient support from management, and work-family disputes have all been repeatedly linked in research to teachers’ mental health issues, anxiety, and depression (Chen et al., 2022; Zhou and Li, 2022b; Zhang et al., 2023; Wang, 2024; Zhong et al., 2025). However, much of the current evidence has focused on occupational risk factors, and little consideration has been compensated to culturally-specific protective aspects that have aided teachers in coping with adversity. The factors of robustness, personal coping mechanisms, and peer support have been frequently explored in Western cultures (Hutchison et al., 2022; Meeks et al., 2023), while more studies include indigenous Chinese cultural concepts that have influenced coping to occupational stress (Zhou and Li, 2022a; Liu L. et al., 2024; Börü et al., 2025). Therefore, limited understanding of how the demands of the occupation (work) relate to the psychological resources embedded in the culture of education (school) in China.

While there has been growing interest in the issue of teacher wellbeing, there are still a number of important research gaps. First, there were limited studies that focused on the pervasiveness of overall psychological distress simultaneously in a representative sample of Chinese teachers (Fang et al., 2023; He et al., 2025). Secondly, there has been limited research that naturally combines occupational stressors, organizational resources, cultural values, coping strategies, and resilience into a holistic explanatory framework that has revealed both direct and indirect mechanisms that affect mental health outcomes (Chen et al., 2022; Wang, 2024; Liao and Hu, 2025). Third, although face concern has been theoretically linked to culturally based coping strategies, it has so far been only marginally explored in terms of its impact on the effectiveness of such strategies. While collectivism (Bellibaş et al., 2024; Börü et al., 2025) and Zhongyong thinking (Zhou and Li, 2022a; Liu L. et al., 2024) have been studied, there has been limited empirical research on the effects of Mianzi (face concern) as a moderator in the teacher mental health model. It has been crucial to overcome these limitations to design culturally relevant interventions that go beyond the traditional workplace stress management approach and include the use of indigenous psychological strengths.

These indigenous constructs were selected because each maps onto a distinct stage of the stress-and-coping process rather than duplicating one another: collectivism represents the value orientation that predisposes teachers toward group-oriented appraisal of adversity; Zhongyong coping represents the behavioral strategy through which that orientation is enacted (balancing, flexibility, and synthesis of competing demands); and Mianzi (face concern) represents the interpersonal-motivational condition that determines how strongly the value-to-coping link is activated. Embedding all three within a single framework therefore allows the model to capture, in sequence, why collectivistic values matter (motivational salience via Mianzi), how they are translated into action (Zhongyong coping), and what psychological resource they ultimately build (resilience) to reduce distress. Grounding the framework in these indigenous constructs, rather than in Western stress-coping models alone, is theoretically important because constructs developed in individualist contexts do not always capture how coping is enacted in relationally oriented, high face-concern cultures such as China’s (Cheng et al., 2014; Kuo, 2013).

This study, therefore, was of the cross-sectional quantitative correlational design, which aimed to investigate the psychological distress of teachers in different educational institutions and in primary, secondary and tertiary educational institutions in 3 municipalities of Jiangsu province. The study specifically sought to determine the incidence of stress, anxiety, depression, and emotional distress; explore the connections between job stress, workplace-family conflict, firm support, and mental anxiety; investigate the orderly mediating role of Zhongyong dealing and cognitive resilience in the connection between collectivistic principles and mental anxiety; and test the moderating role of Mianzi in this association. How do Chinese teachers’ emotional trouble, anxiety, stress, and depressive symptoms relate to typical responsibilities, employment-family conflict, collectivistic values, Zhongyong coping, mental toughness, and Mianzi? This investigation therefore seeks to: (1) recognize the prevalence of psychological health problems among teachers in China; (2) explore associations between occupational stressors, organizational support and psychological distress; (3) examine the mediation effect sequence of Zhongyong coping and psychological resilience; and (4) test the role of Mianzi in the proposed arbitration paths. The findings offer a more holistic view of mental health among teachers, which includes occupational and culturally specific psychological factors, and offer evidence to support culturally responsive education policies and psychological support programs in China in the future.

### 1.2 Theoretical and empirical foundation

Empirical studies confirmed that teachers were one of the occupational groups at high risk of psychological distress due to the growing responsibilities in teaching, administrative burden, and changes in educational expectations. The incidence and causes of mental health problems were, however, highly context-specific, both in terms of geography, level of education, and methodology used. There were more and more studies recently that added organizational, psychological, and cultural factors to the more comprehensive explanatory frameworks, with a limited amount of evidence that integrated indigenous Chinese cultural constructs. The existing studies were thus useful in the sense of gaining insight into teacher wellbeing, but also showed significant gaps in the conceptual and empirical dimensions that merited further research.

Several studies evaluated the status among Chinese teachers with the aid of standardized psychological instruments. In a broad poll of elementary and secondary school teachers in Dongguan, He et al. (2025) found that 19.3% of educators had anxiousness, 34.7% had a mental illness, and 74.5% had fatigue. While female instructors were more likely to experience intellectual weariness, elementary school teachers and those with greater academic success were less likely to experience depression. Likewise, Fang et al. (2023) surveyed teachers in the economically disadvantaged areas in Ningxia Province, which revealed that about a quarter of the teachers had potential mental health issues based on the Symptom Checklist-90. Teachers had significantly lower psychological functioning in several areas, such as depression, anxiety, obsessive-compulsive symptoms, hostility, psychotic symptoms, and somatization, compared to national norms. The mental health status was significantly different across age bands and marital bands, with younger and unmarried teachers reporting relatively favorable mental health status.

Studies done on a global scale also validated the prevalence of psychological distress among educational professionals. In an American university, Meeks et al. (2023) reported that almost one-third of faculty and staff reported suffering from terrible and expectionally extreme tension, nervousness, and sorrow. The educational population has been even more complex because of the differences in coping and barriers to professional mental healthcare that were exacerbated. Similarly, anxiety symptoms and psychological distress were much higher in Canadian school staff during the COVID-19 pandemic than national population estimates, according to Hutchison et al. (2022). There was a significant and strong relationship between greater direct contact with students and psychological symptoms of anxiety, reflecting the increased psychological burden from occupational exposure. Taken as a whole, these inquiries showed that psychological stress was a global issue for educators that was present in both contexts.

In addition to prevalence estimation, several studies investigated occupational factors influencing teachers’ mental health. In Shanxi Province, Zhang et al. (2023) studied the difference in stress levels between teachers and non-teaching education workers and found that the teachers showed significantly higher stress than the non-teaching education workers. Stress for primary school teachers was predicted independently by larger class sizes, while the most important risk factor for secondary school teachers was greater weekly teaching hours. A similar study by Wang (2024) showed that With modeling structure equations, high school teachers’ perceived support from management, job involvement, and mental health were all negatively impacted by workload. This was consistent with the study by Zhou and Li (2022b), which revealed that for each new and seasoned preschool teachers, the tension between work and family had a major detrimental impact on emotional tiredness. Their study also revealed that both continuance commitment and affective commitment protected this relationship among both groups of teachers, but only affective commitment protected experienced teachers. These studies, taken together, clearly indicated that occupational demands were basic antecedents of teacher psychological distress.

Several investigations identified the organizational resources as equally important protective factors. Chen et al. (2022) examined elementary school teachers in rural China by demonstrating how perceived support from management affected the relationship between job fulfillment and fatigue. Work participation and job fulfillment were significantly positively correlated with company backing, while burnout was significantly negatively correlated with it. Likewise, Liao et al. (2023) found three psychological need-thwarting profiles in Chinese teachers and teachers who had lower psychological need frustration had lower psychological distress and higher perceived administrative support than those in higher-risk profiles. These results all suggested that supportive organizational environments had a significant positive impression on psychological wellbeing and reduced the undesirable effects of occupational demands.

Since they can reduce work-related anxiety, household and societal assets have also received a lot of focus in the research. Zhong et al. (2025) demonstrated that family solidarity significantly mediated the relationship between perceived demands on time and workplace-family disputes in reducing teachers’ distress in a sample of more than three thousand Chinese teachers. A greater degree of family assistance were associated with a lower prevalence of mood disorders, workplace-family disputes, and reported time scarcity among teachers. The findings were consistent with organizational support research, which showed that resources outside of the workplace also significantly impacted psychological wellbeing. These investigations combined indicate the recognition that organizational and interpersonal resources worked together to impact teachers’ mental health.

Psychological resilience and adaptive coping were explored as mechanisms linking personal resources to better psychological outcomes and became the focus of recent studies. Liao and Hu (2025) constructed a Conservation of Resources-based sequential mediation model in Chinese university teachers and revealed that mindfulness had a significant impact on enhancing resilience and cognitive reappraisal, which further facilitated stress self-management. Mindfulness was related to stress management through two mechanisms: Resilience and cognitive reappraisal, and explained a substantial portion of the variation in adaptive ability. Likewise, Zhou and Li (2022a) found that University students’ resilience was significantly improved by cognitive reappraisal, positive affect, and their subsequent combination, with the latter pathway leading to the most noticeable results. These results, even if taken out of the school system, pointed to the important psychological processes by which culturally based cognition enhanced resilience and adaptive functioning.

Empirical attention also grew toward collectivistic values and culturally embedded psychological processes that impact wellbeing. Collectivism was shown to positively impact teachers’ wellbeing via principals’ social justice leadership by Börü et al. (2025). They concluded that collectivistic orientations were indirectly related to psychological outcomes through strengthening supportive organizational leadership. Similarly, the verdicts of Bellibaş et al. (2024) specified that the wellbeing of teachers completely mediated the association between paternalistic leadership and organizational commitment, and that the collectivistic school culture was a positive booster of these indirect relationships. Together, these results indicated that collectivistic orientations were useful situational resources that were associated with better organizational performance and psychological health, not just a cultural choice.

Information on the nature of Zhongyong thinking further reinforced the argument for using indigenous Chinese cultural constructs in occupational mental health studies. Liu L. et al. (2024) found that negative thinking of the Zhongyong type was an important indicator of anxiety in Chinese university students and that conscientiousness was an active moderator, making the negative effect even stronger. Likewise, Zhou and Li (2022a) determined positive correlations between Zhongyong thinking and resilience, while Liu M. et al. (2024) drew a picture of psychological mechanisms between workplace ostracism and unproductive work practices with an acceptable degree of self-worth bridging. Despite the different populations and outcomes examined, all of these investigations found culturally based cognitive styles to be related to psychological resources and contextual conditions and how these factors relate to behavioral and emotional outcomes.

There was also methodological evidence for using strong psychometric tools and modeling techniques to explore teacher mental health. A large sample of Chinese teachers (*N* > 9000) was used by Cao et al. (2023) to thoroughly test the DASS-21, which showed good internal consistency, convergent validity, and longitudinal measurement invariance, although some problematic items were found for the stress scales. As the reviewed research progresses, the use of structural equation modeling, mediation analysis, moderated mediation, latent profile analysis, and confirmatory factor analysis has increased in the studies that studied complex and multiple relationships among occupational, psychological, organizational, and cultural variables.

The overall findings of the literature review indicated that organizational support, resilience, family support, and adaptive coping were important resources that buffer psychological distress and that occupational demands were consistently shown to exacerbate psychological distress. However, there were still significant research opportunities. There have been few studies that have investigated prevalence, occupational stressors, organizational support, collectivistic values, Zhongyong coping, resilience and psychological distress within a single integrated model that tested the moderated-mediation model. Furthermore, this moderation effect of Mianzi (face concern) has been studied very little empirically, although its central role in Chinese culture is evident. As a result, much of what was known about the relationship between indigenous cultural resources and occupational stressors, and their impact on mental health among teachers, was limited. To overcome these limitations, the current study combined occupational and culturally specific protective factors into a holistic theoretical model and assessed them in a sample of teachers in a variety of educational contexts in the Chinese province of Jiangsu, which enriched the existing literature on teacher psychological wellbeing.

### 1.3 Hypotheses formulation

This study’s theoretical framework is based on the Job Demands–Resources (JD–R) Theory (Bakker and Demerouti, 2007), the Conservation of Resources (COR) Theory (Hobfoll, 1989), and the Social Cognitive Theory (SCT) (Bandura, 1986). Together, these views provide insights into the processes through which occupational demands create psychological strain, through which personal and contextual resources buffer against depletion of resources, and through which culturally embedded cognitive and behavioral processes enable adaptation to stressful working conditions. A synthesis of these complementary theoretical lenses offers a comprehensive lens for understanding the psychological distress of Chinese teachers with consideration for organizational factors, individual factors, and culturally unique coping mechanisms.

Teaching has been demanded quite a lot of emotionally, cognitively and administratively, and sometimes the resources needed for teaching have not been provided in the teachers’ personal or organizational capacity (Chang, 2009). In the current context, burden and tension between job and family have been significant needs that drain psychological energy, while organizational support has been an important contextual resources that assist teachers in the management of their professional needs and wellbeing (Mansour and Tremblay, 2016; Nwoko et al., 2023). It has been anticipated that a history of excessive demands that has not been supported by the organization has caused greater susceptibility to depression, anxiety, stress and psychological distress overall (Ghasemi, 2025). In the rapidly evolving landscape of education, these factors have been especially pertinent as the demands of accountability, administration, and family responsibilities increase occupational stress. Therefore, increased psychological symptoms have been likely to be found in a significant number of teachers, and higher workload and work–family conflict (with low organizational support) have been expected to be related to poorer mental health. Therefore, the following hypotheses have been put forward:

*H1*: Higher occupational stressors—workload and work–family conflict—are significantly positively associated with psychological distress among Chinese teachers, whereas higher perceived organizational support is significantly negatively associated with distress.

In addition to the occupational demands, culturally embedded personal resources have influenced the perception and management of stressful situations and thus affect psychological adjustment (Cheng et al., 2014). Collectivistic values focus on social harmony, social responsibility, and problem-solving together, giving people cognitive orientations that help them adapt to adversity (Kuo, 2013). In the Chinese culture, these values have been similar to the concept of Zhongyong coping, which involves balancing, flexibility, moderation and synthesizing multiple perspectives to respond to stressful incidents (Yao et al., 2024; Guo et al., 2024). Strong endorsement of collectivistic values has been expected to be linked with teachers’ higher frequencies of using Zhongyong coping in response to work stressors, as well as lower emotional reactivity and higher levels of constructive adapting (Li et al., 2023). With repeated use of such adaptive coping mechanisms, a person builds up psychological resources that increase resilience and, ultimately, reduce psychological distress. Thus, the effects of collectivistic values on psychological wellbeing have been hypothesized to be mediated by culturally-informed coping and resilience processes in a sequence. So the following hypotheses have been proposed:

*H2*: The relationship between collectivistic cultural values and psychological distress is sequentially mediated by Zhongyong coping and psychological resilience, such that collectivistic values are positively associated with Zhongyong coping, Zhongyong coping is positively associated with resilience, and resilience is negatively associated with psychological distress.

The relationship between collectivistic values and coping behavior has not been uniform across people, as there has been a possibility of adaptive coping (Chun et al., 2006). Faced with the significance of social reputation, interpersonal respect and harmonious social relationships, concern has been considered an aspect of motivation with cultural meaning that has enhanced or hindered behaviors related to collectivistic norms, which has been what Zhou and Zhang (2024) have named. This advises that the positive consequence of collectivistic values on Zhongyong coping has been hypothesized to be greater the higher the face concern. The level of coping with the challenge of having to adjust to the Chinese context of collectivism is also expected to influence the indirect pathway between collectivism and mental health outcomes, as coping with the challenge of collectivism has been likely to facilitate resilience, and psychological distress has been likely to be reduced (Cui et al., 2022). As such, face concern has been hypothesized to serve as a moderator between cultural values and coping as well as a borderline state on the overall indirect pathway.

*H3*: Mianzi (face concern) moderates the collectivistic-values-to-Zhongyong-coping path (the first stage of the mediation model) and, consequently, the overall conditional indirect (moderated-mediation) effect of collectivistic values on psychological distress through Zhongyong coping and resilience, such that the protective indirect pathway is stronger among teachers with higher face concern.

In general, the three hypotheses presented above jointly combine the organizational resources, culturally embedded values, adaptive coping, resilience, and psychological distress, with occupational demands, and relate them into a moderated-mediation model.

## 2 Research methodology

This study examined the incidence of psychological disorders among Chinese teachers and tested a moderated-mediation model of the relationship between psychological disorders, culturally appropriate coping mechanisms, and job stressors using a cross-sectional statistical correlational research methodology. In order to estimate the prevalence, test several associated hypotheses, and obtain a reliable and statistically valid sample of a large and geographically distributed teacher community, a cross-sectional survey methodology was employed.

This research took place in three cities, with different socioeconomic and educational backgrounds: Nanjing (the provincial capital with urban and high resource schools), Suzhou (with economically developed suburban district schools), and Xuzhou (with economically less-developed semi-rural district schools) in northern Jiangsu province, China. The tri-city sampling frame was chosen to achieve good coverage of the variance in school resourcing, teacher workload and cultural-regional variation across a single province. The primary schools, junior high, senior high and vocational/tertiary colleges registered by the Jiangsu Provincial Department of Education were selected as the respondents.

A stratified random sampling technique was used to split into two strata—school level (primary, secondary, tertiary) and municipality (Nanjing, Suzhou, Xuzhou)—to get nine strata. For each stratum, schools and teachers were randomly selected based on the size of each stratum. The necessary sampling size was determined to be 600.25 using Cochrane’s formula for an infinite population, which was rounded to 600. To account for an anticipated 20% non-response, the final target sample was changed to 750. The number of 750 in-service teachers was thus targeted, and recruitment was continued until the number reached 750 across the 8 latent variables in the planned structural model and 9 strata. The sampling frame did not include substitute or part-time teachers, administrators who do not teach, nor teachers who were on extended medical or maternity leave at the time of data collection.

In addition to the Cochran’s-formula estimate used for the prevalence component, an a priori power analysis for the planned multiple-regression and moderated-mediation tests was conducted using G*Power 3.1 (linear multiple regression, fixed model, R^2^ deviation from zero). Assuming a small-to-medium effect size (*f*^2^ = 0.04), α = 0.05, power (1−β) = 0.95, and up to eight predictors (including covariates), the minimum required sample was approximately 216 participants. Because the multi-stage (municipality × school-level) stratified design introduces clustering, a design effect (DEFF) of 1.5 was applied (DEFF = 1 + (m–1)ρ, with an assumed intraclass correlation ρ≈.02 and average cluster size m ≈ 25), yielding a design-adjusted minimum requirement of approximately 324 participants. The final achieved sample of 750 (after accounting for the 20% anticipated non-response/invalid-questionnaire allowance) therefore exceeded both the prevalence-based and the power-based sample size requirements.

A standardized, self-administered questionnaire with eight sections that represented the eight study variables served as the instruments. Occupational workload was measured using the Job Demands section of Karasek et al.’s (1998) Job Content Questionnaire (JCQ). The Work-Family Conflict Scale, created by Netemeyer et al. (1996) and extensively verified across the Chinese population, was used to measure work-family conflict (Lu et al., 2008). The eight-item Survey of Perceived Organizational Support (SPOS) by Eisenberger et al. (1986) and the brief version validated by Eisenberger et al. (1997) were used to measure perceived organizational support. Chinese Culture Connection (1987) has developed and assessed the cultural orientation of the Chinese using the individualism–collectivism dimension. The Zhongyong dialectical coping mediated between the two variables in the study was measured by the Zhongyong Coping Scale developed and validated by Wu and Lin (2005). Mianzi (face concern) was measured with an adapted Face Concern Scale proposed by Zhang et al. (2011). Psychological resilience was assessed by the Chinese version of the Connor–Davidson Resilience Scale (CD-RISC-25), which was created by Connor and Davidson (2003) and validated for the Chinese population by Yu and Zhang (2007). The outcome variable was psychological distress measured by the 21-item Depression Anxiety Stress Scale (DASS-21) developed by Lovibond and Lovibond (1995), and the Kessler Psychological Distress Scale (K10) developed by Kessler et al. (2002) to validate the DASS-21. All the instruments were translated in a forward–backward translation procedure that was done with bilingual experts in accordance with cross-cultural adaptation guidelines (Beaton et al., 2000).

Fifty teachers participated in a pre-test of the translated instrument to evaluate its internal consistency, cultural appropriateness, and intelligibility (Nunnally and Bernstein, 1994). Ten weeks were spent gathering the data. After obtaining ethical approval from the institutional review board of the researchers and formal permission from the school principals of the participating schools, an electronic questionnaire link created on the platform of Wenjuanxing, a Chinese survey platform widely used in China, was sent to the teachers using the school coordinators, and the paper-based questionnaire was sent to the schools with limited internet access. The survey was anonymous and voluntary, and informed consent was obtained online before entry into the survey. Non-respondents were sent reminder messages every 2 weeks. Data were checked weekly for completeness, and respondents with more than 10% missing were replaced by additional random sampling within the appropriate stratum until the 750 valid respondents were obtained. Participants, whether or not they responded to the question, had a mental health referral list attached to the closing page of the questionnaire, which provided details of contacts for the provincial counseling hotlines. The data were analyzed in four steps within SPSS (version 27) and Mplus (version 8.7). Descriptive statistics and prevalence rates (percentage of respondents with scores above clinical cutoffs on DASS-21 and K10) were computed and compared across strata, with chi-square tests. The proposed conceptual framework illustrating the relationships among occupational stressors, collectivistic values, Zhongyong coping, resilience, Mianzi, and psychological distress is presented in Figure 1. The hypothesized sequential mediation model was estimated by the following set of regression equations:

**FIGURE 1:**
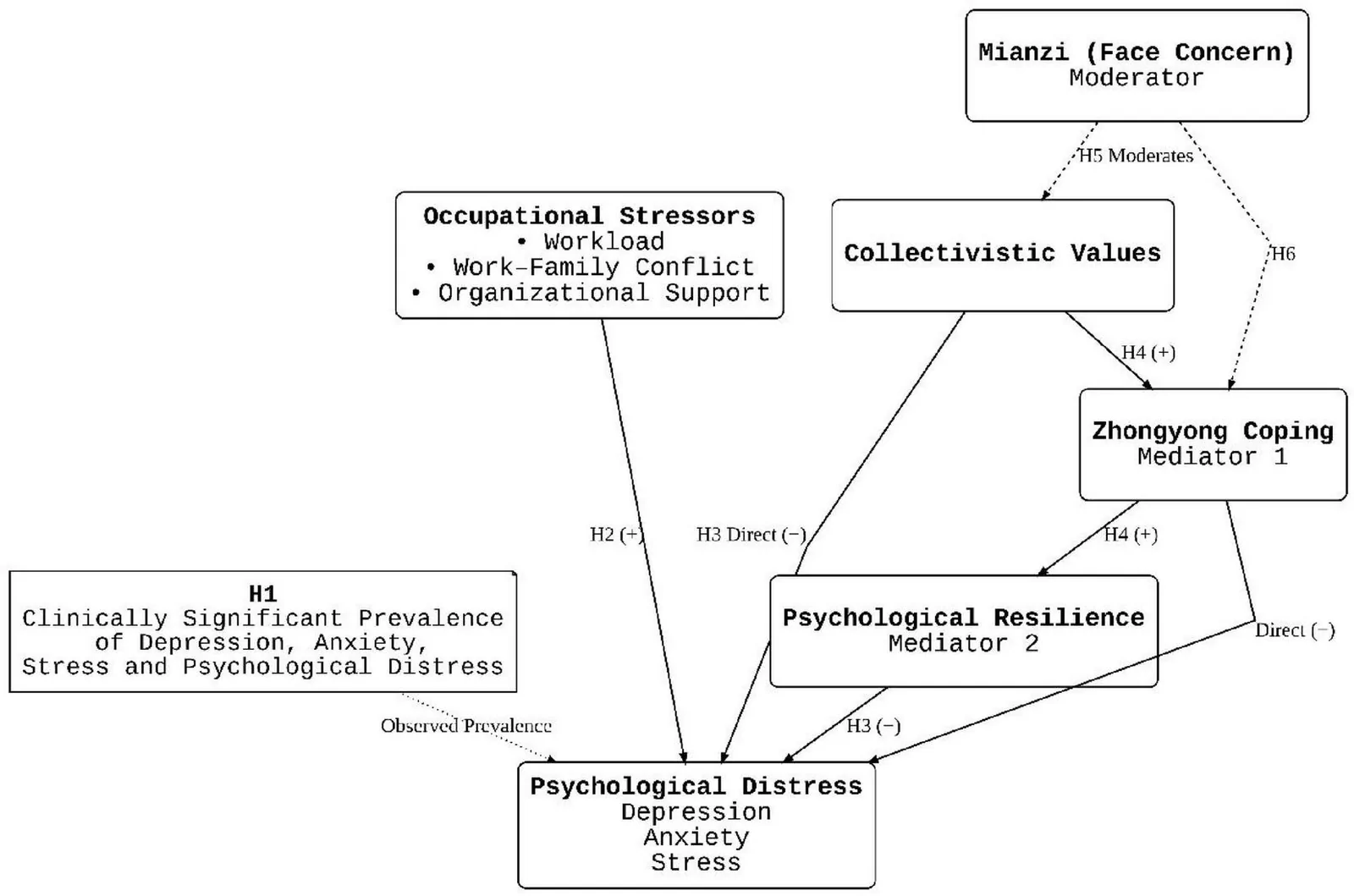
Conceptual framework diagram. Single-headed arrows denote hypothesized directional (regression) paths; the dashed arrow from Mianzi (Face Concern) to the Collectivism→Zhongyong Coping path denotes the hypothesized first-stage moderation effect (a4). CV, Collectivistic Values; ZC, Zhongyong Coping; RES, Psychological Resilience; DIS, Psychological Distress.

Regarding missing data: no dataset used in the final analyses contained missing values on the study variables, because the quality-control procedure described above (weekly completeness checks, with respondents exceeding 10% missingness excluded and replaced through additional stratified random sampling) was applied prospectively during data collection until 750 fully valid, complete responses were obtained. Consequently, no *post-hoc* imputation technique (e.g., mean imputation, multiple imputation, or FIML) was required or applied.


$$M_{1}=\alpha_{1}+a_{1}\,X+\overset{k}{\underset{i=1}{\sum }}\gamma_{i}\,C_{i}+\varepsilon_{1}$$

Where, *M*_1_ = Zhongyong Coping, *X* = Collectivistic Values; *C*_*i*_ = Covariates (Workload, Work–Family Conflict, Organizational Support); *a*_1_ = Path coefficient; ε_1_ = Error term


$$M_{2}=\alpha_{2}+a_{2}\,X+b_{1}\,M_{1}+\overset{k}{\underset{i=1}{\sum }}\delta_{i}\,C_{i}+\varepsilon_{2}$$

Where, *M*_2_ = Psychological Resilience; *b*_1_ = Effect of Zhongyong Coping on Resilience; *a*_2_ = Direct effect of Collectivistic Values on Resilience


$$Y=\alpha_{3}+c^{{}^{\prime }}\,X+c_{1}\,M_{1}+b_{2}\,M_{2}+\overset{k}{\underset{i=1}{\sum }}\lambda_{i}\,C_{i}+\varepsilon_{3}$$

Where, *Y* = Psychological Distress; *c*′ = Direct effect of Collectivistic Values; *c*_1_ = Direct effect of Zhongyong Coping; *b*_2_ = Effect of Resilience

The sequential mediation effect was calculated as


$$\mathrm{Indirect}\,\mathrm{Effect}=a_{1}\times b_{1}\times b_{2}$$

For moderated mediation, Hayes’ PROCESS Model 7 was used, which specifies moderation of the first-stage path (Collectivism → Zhongyong Coping) only; the second-stage and direct paths (b1, b2, c’) are held constant across levels of the moderator, consistent with the conditional-indirect-effect pattern reported in Table 1:


$$M_{1}=\alpha_{4}+a_{1}\,X+a_{3}\,W+a_{4}\,\left(X\times W\right)+\overset{k}{\underset{i=1}{\sum }}\phi_{i}\,C_{i}+\varepsilon_{4}$$

**TABLE 1:** Conditional indirect effects at levels of Mianzi (face concern).

Mianzi Level	Conditional a1 (CV→ZC)	Conditional indirect effect	Boot LLCI	Boot ULCI
–1 SD (low face concern)	0.266	–0.025	–0.039	–0.014
Mean	0.351	–0.033	–0.047	–0.023
+1 SD (high face concern)	0.435	–0.041	-0.056	–0.027

where, *W* = Mianzi (Face Concern); *X*×*W* = Interaction term; *a*_4_ = Moderation coefficient

### 2.1 Conditional effect of collectivistic values


$$a_{\text{conditional}}=a_{1}+a_{4}\,W$$

### 2.2 Conditional indirect effect


$$\theta \,\left(W\right)=\left(a_{1}+a_{4}\,W\right)\times b_{1}\times b_{2}$$

Where, θ(*W*) = Conditional indirect effect at a given level of Mianzi

### 2.3 Index of moderated mediation


$$\text{IMM}=a_{4}\times b_{1}\times b_{2}$$

Furthermore, multigroup structural equation modeling analysis was carried out to examine the invariance of the structural relationships among subgroups by applying the chi-square difference test and a CFI difference of less than 0.01 to prove both measurement and structural invariance.

## 3 Results

The study was conducted with 750 teachers using stratified random sampling: three municipalities (Nanjing, Suzhou, and Xuzhou) and three education levels (primary, secondary, and tertiary). The total respondents were mostly female (70.4%) and male (29.6%), as shown in Table 2. There was nearly equal representation across the three municipalities, with about one-third of participants recruited from each municipality. Similarly, there was equal representation in schools across all levels with matched comparisons among primary, secondary and tertiary institutions. The average age and teaching experience meant a highly adult and professionally experienced teaching workforce suitable for studying occupational stress and mental health outcomes.

**TABLE 2:** Sample demographics (*N* = 750).

Variable	Category	n	%
Gender	Female	528	70.4
	Male	222	29.6
Municipality	Nanjing	252	33.6
	Xuzhou	249	33.2
	Suzhou	249	33.2
School level	Primary	250	33.3
	Secondary	250	33.3
	Tertiary	250	33.3
School type	Public	620	82.7
	Private	130	17.3
Age (years)	Mean (SD)	37.6	(7.6)
Teaching experience (years)	Mean (SD)	15.2	(7.6)

During the psychometric assessment, it was found that all the measuring tools had internal consistency reliability at an acceptable level (Table 3). The results of these tests showed that all constructs were measured consistently and reliably, which in turn confirmed the quality of their suitability for correlational, mediation, moderation and structural analyses. Table 4 also showed moderate levels of workload, work-family conflict, collectivist values, organizational support, Zhongyong coping, face concern, resilience and psychological distress within the sample, which provided sufficient variance for hypothesis testing.

**TABLE 3:** Scale reliability (Cronbach’s α).

Scale	No. of Items	Cronbach’s α
Workload (JCQ demands)	5	0.818
Work–family conflict	5	0.820
Organizational support (SPOS)	8	0.878
Collectivistic values (CVS)	6	0.842
Zhongyong coping	13	0.926
Mianzi/face concern	10	0.905
Psychological resilience (CD-RISC)	25	0.955
DASS-21 depression	7	0.849
DASS-21 anxiety	7	0.852
DASS-21 stress	7	0.852
K10 psychological distress	10	0.898

**TABLE 4:** Descriptive statistics for composite scale scores.

Variable	Mean	SD	Min	Max
Workload	12.49	4.25	5	20
Work–family conflict	19.90	7.61	5	35
Organizational support	32.16	11.84	8	56
Collectivism	21.13	7.68	6	36
Zhongyong coping	45.42	16.17	13	78
Mianzi (face concern)	29.81	10.25	10	50
Psychological resilience	50.18	24.60	0	100
DASS total	62.81	29.37	0	126
K10 total	30.04	10.16	10	50

Cultural values → Zhongyong coping → resilience → distress, controlling for workload, work–family conflict, and organizational support.

The prevalence analysis indicated that there were significant mental health issues with Chinese teachers. Table 5 indicates that only 17.9% of all participants had normal depression levels, with 73.4% of them having moderate to extremely severe depression levels. The pattern was even more troubling for anxiety, as 80.5% of teachers answered that they felt moderate to extremely severe anxiety and over half (54.8%) fell into the extremely severe category. Stresses were generally higher in the past and still significant, with 56.8% identifying as having moderate or more severe stressors. In line with these, according to the K10 scale, 52.5% of the respondents had very high psychological distress, and 14.8 and 13.2% had high and moderate psychological distress, respectively (Table 6). The prevalence of depression was also significantly different among municipalities [χ^2^(2) = 10.65, *p* = 0.005]. Hence, teacher teachers in lower-resource municipalities reported a higher psychological load.

**TABLE 5:** Prevalence of DASS-21 symptom severity.

Scale	Normal	Mild	Moderate	Severe	Extremely severe
Depression	17.9% (*n* = 134)	8.8% (*n* = 66)	22.4% (*n* = 168)	17.5% (*n* = 131)	33.5% (*n* = 251)
Anxiety	13.2% (*n* = 99)	6.3% (*n* = 47)	15.2% (*n* = 114)	10.5% (*n* = 79)	54.8% (*n* = 411)
Stress	33.9% (*n* = 254)	9.3% (*n* = 70)	18.8% (*n* = 141)	19.5% (*n* = 146)	18.5% (*n* = 139)

**TABLE 6:** K10 psychological distress categories.

Category	n	%
Low distress (10–19)	146	19.5
Moderate distress (20–24)	99	13.2
High distress (25–29)	111	14.8
Very high distress (30–50)	394	52.5

Chi-square test: Moderate-or-above depression prevalence differed significantly by municipality, χ^2^(2) = 10.65, *p* = 0.005—consistent with Xuzhou’s lower-resource school context.

Table 7 showed satisfactory construct validity, with standardized factor loadings mostly above recommended thresholds; discriminant validity is reported in Supplementary Tables S1, S2. The standardized factor loading values were mostly above the recommended values (Figure 2).

**TABLE 7:** Measurement model—single-factor CFA-style loading summary.

Construct	No. items	Min loading	Max loading	Variance explained	AVE	CR
Workload	5	0.742	0.781	0.579	0.579	0.873
Work–family conflict (WFC)	5	0.746	0.796	0.581	0.581	0.874
Organizational support	8	0.721	0.764	0.540	0.540	0.904
Collectivism	6	0.736	0.757	0.559	0.559	0.884
Zhongyong coping (novel)	13	0.701	0.763	0.529	0.529	0.936
Mianzi/face concern (novel)	10	0.707	0.747	0.538	0.538	0.921
Resilience	25	0.665	0.732	0.484	0.484	0.959
Psychological distress	21	0.595	0.664	0.389	0.389	0.930

**FIGURE 2:**
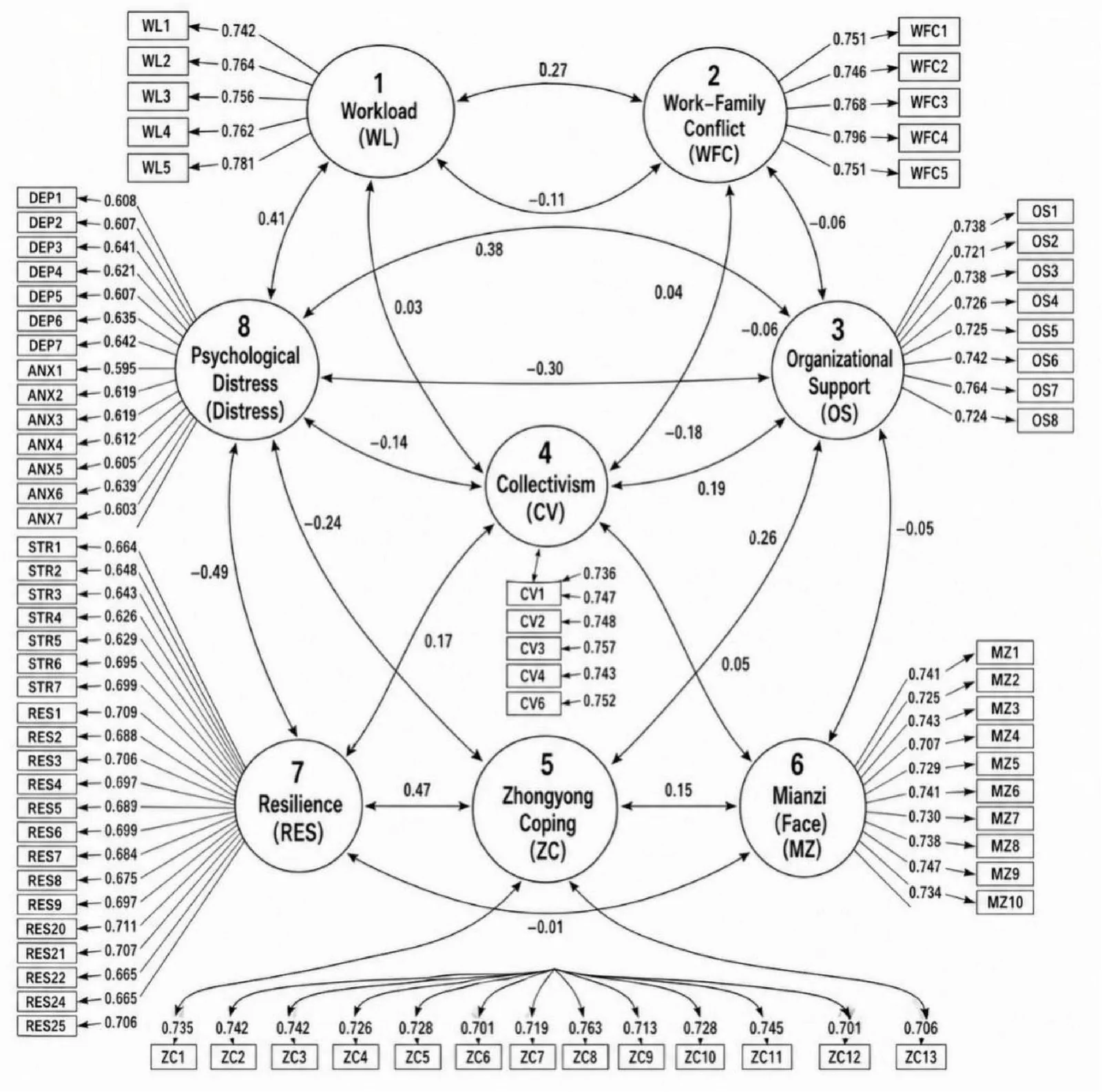
Measurement model. Values on measurement paths are standardized factor loadings (λ); all loadings were significant at *p* < 0.001. Ellipses represent latent constructs; rectangles represent observed indicators/parcels.

The hypothesized relationships between occupational stressors, cultural variables, resilience and psychological distress were supported by correlation analysis (Table 8). The correlation heatmap showing the associations among the study variables is presented in Figure 3. Workload (*r* = 0.41) and work-family conflict (*r* = 0.38) were found to have the highest positive relationships with psychological distress, while organizational support had a negative correlation (*r* = –0.30). Among the protective correlates of distress, coping with the experience was strongest, with coping of Zhongyong (*r* = –0.41) and psychological resilience (*r* = –0.49). Collectivistic values significantly and positively correlated with Zhongyong coping (*r* = 0.36) and resilience (*r* = 0.17) and had a small negative correlation with distress (*r* = –0.14). The results suggest that the psychological distress and adverse mental health outcomes have been mediated by occupational demands, while culture and psychological resources mitigate the impact of occupational demands.

**TABLE 8:** Correlation matrix among study variables (Pearson’s *r*).

	Workload	WFC	Org. Support	Collectivism	Zhongyong	Mianzi	Resilience	Distress
Workload	1.00	1.00	1.00	1.00	1.00	1.00	1.00	1.00
Work–family conflict	0.27							
Org. Support	–0.11	–0.06						
Collectivism	0.03	0.04	–0.06					
Zhongyong coping	–0.21	–0.18	0.19	0.36				
Mianzi (Face)	0.06	0.03	–0.05	0.28	0.15			
Resilience	–0.24	–0.21	0.26	0.17	0.47	–0.01		
Distress	0.41	0.38	–0.30	–0.14	–0.41	0.05	–0.49	

**FIGURE 3:**
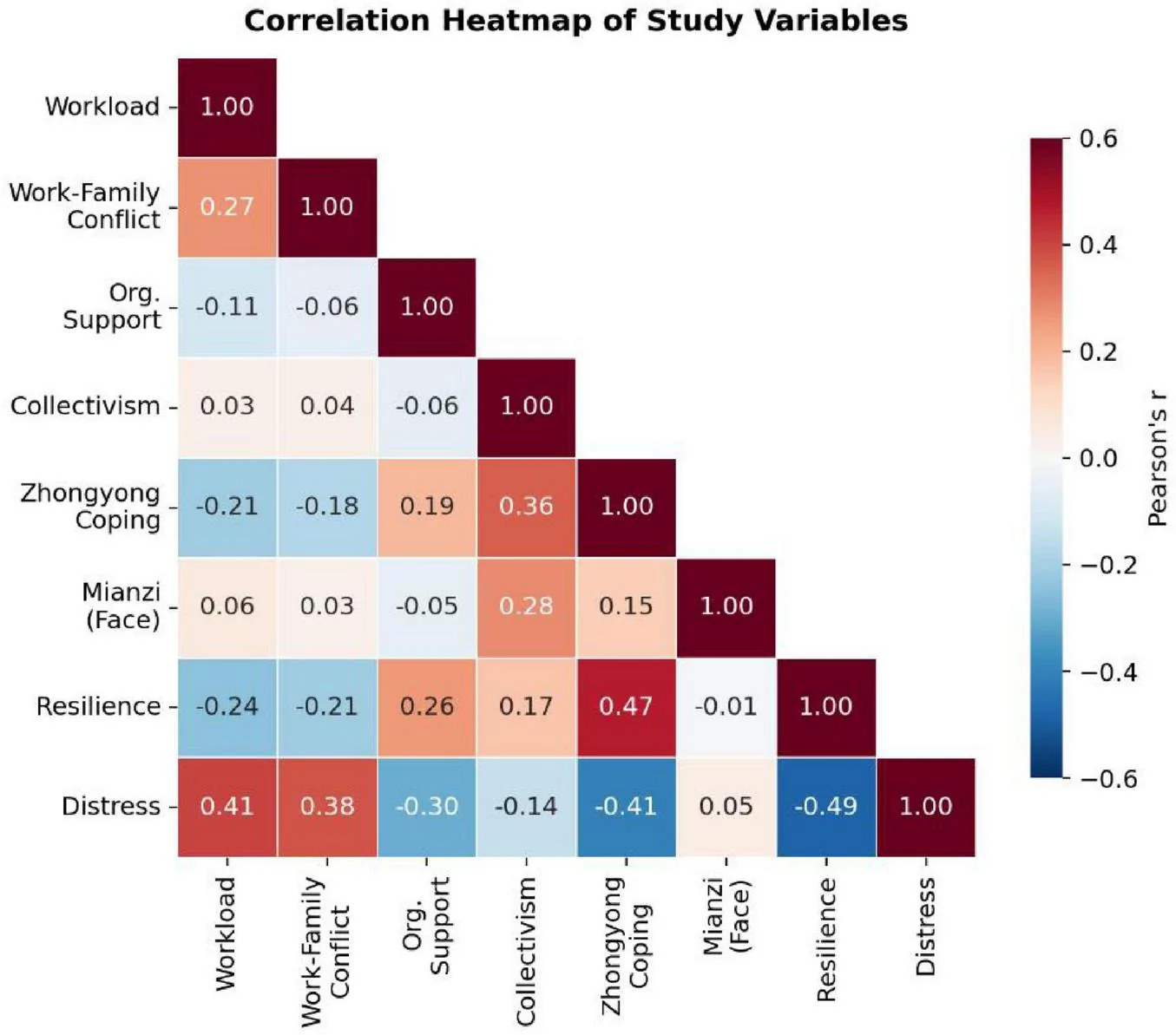
Correlation heatmap. Cell values are Pearson correlation coefficients (r) among study variables (see Table 8 for exact values); darker shading indicates stronger association.

Results presented in Table 9 indicated collectivistic values significantly predicted Zhongyong coping (β = 0.379, *p* < 0.001), which in turn favorably influenced resilience (β = 0.369, *p* < 0.001). The proposed sequential mediation pathway linking collectivistic values, Zhongyong coping, psychological resilience, and psychological distress is illustrated in Figure 4. The psychological distress was also predicted to have a favorable negative association with resilience (β = –0.257, *p* < 0.001) and also with Zhongyong coping (β = –0.139, *p* < 0.001). These findings suggest partial mediation as collectivistic values remained significantly and negatively associated with distress (β = –0.075, *p* = 0.014) even when both mediators were included. These findings confirm that collectivist values have a direct favorable influence on decreasing psychological distress and also have a positive effect on improving culturally appropriate coping and resilience.

**TABLE 9:** Sequential mediation model—standardized regression paths.

Path	B (std.)	SE	*t*	*p*
a1: Collectivism → Zhongyong coping	0.379	0.032	11.77	<0.001
b1: Zhongyong coping → resilience	0.369	0.035	10.41	<0.001
b2: Resilience → distress	–0.257	0.033	–7.84	<0.001
c1: Zhongyong coping → distress (direct)	–0.139	0.034	–4.10	<0.001
c’: Collectivism → distress (direct, controlling mediators)	–0.075	0.030	–2.47	0.014
c: Collectivism → distress (total effect)	–0.179	0.030	–5.99	<0.001

Cultural values → Zhongyong coping → resilience → distress, controlling for workload, work–family conflict, and organizational support.

**FIGURE 4:**
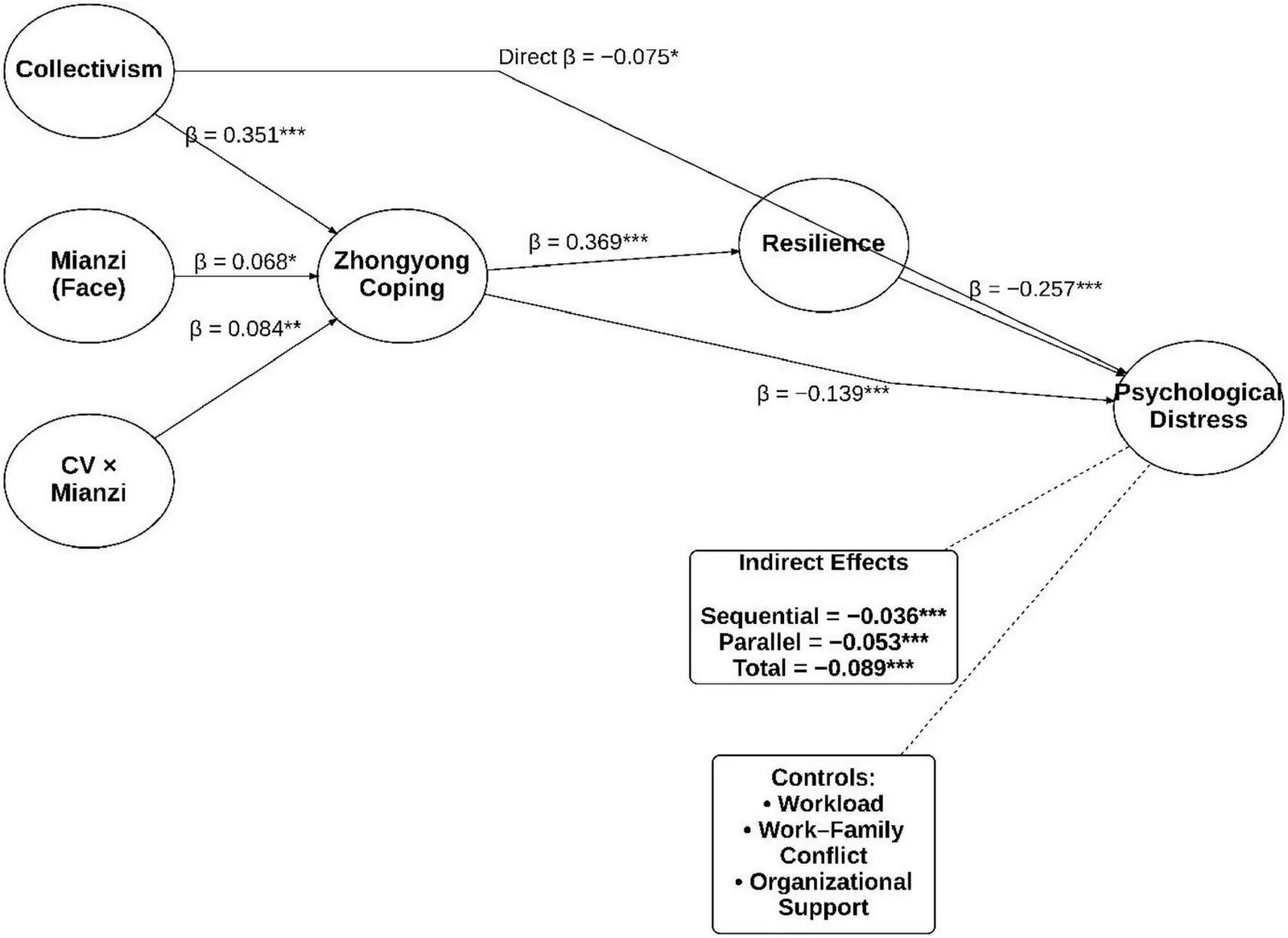
Pathway diagram. Values shown are standardized path coefficients (β); *p < 0.05, **p < 0.01, ***p < 0.001 (see Table 9 for full statistics).

All indirect pathways were statistically significant via bootstrapping (Table 10). The conditional indirect effects of collectivistic values on psychological distress through Zhongyong coping and psychological resilience at different levels of Mianzi are presented in Figure 5. The sequential indirect effect of collectivistic values through Zhongyong coping and resilience to psychological distress was significant (β = –0.036, 95% CI [–0.049, –0.024]). Likewise, the parallel indirect pathway involving Zhongyong to coping alone was also significant (β = –0.053, 95% CI [–0.080, –0.028]). The total indirect effect was also statistically significant (β = –0.089, 95% CI [–0.118, –0.062]), indicating that culturally embedded coping processes have been significant factors in lessening psychological distress when collectivistic values have been taken into account.

**TABLE 10:** Indirect effects.

Effect	Estimate	Boot LLCI	Boot ULCI	Significant
Sequential indirect (CV → ZC → RES → DIS)	–0.036	–0.049	–0.024	Yes
Parallel indirect (CV → ZC → DIS)	–0.053	–0.080	–0.028	Yes
Total indirect effect	–0.089	–0.118	–0.062	Yes

**FIGURE 5:**
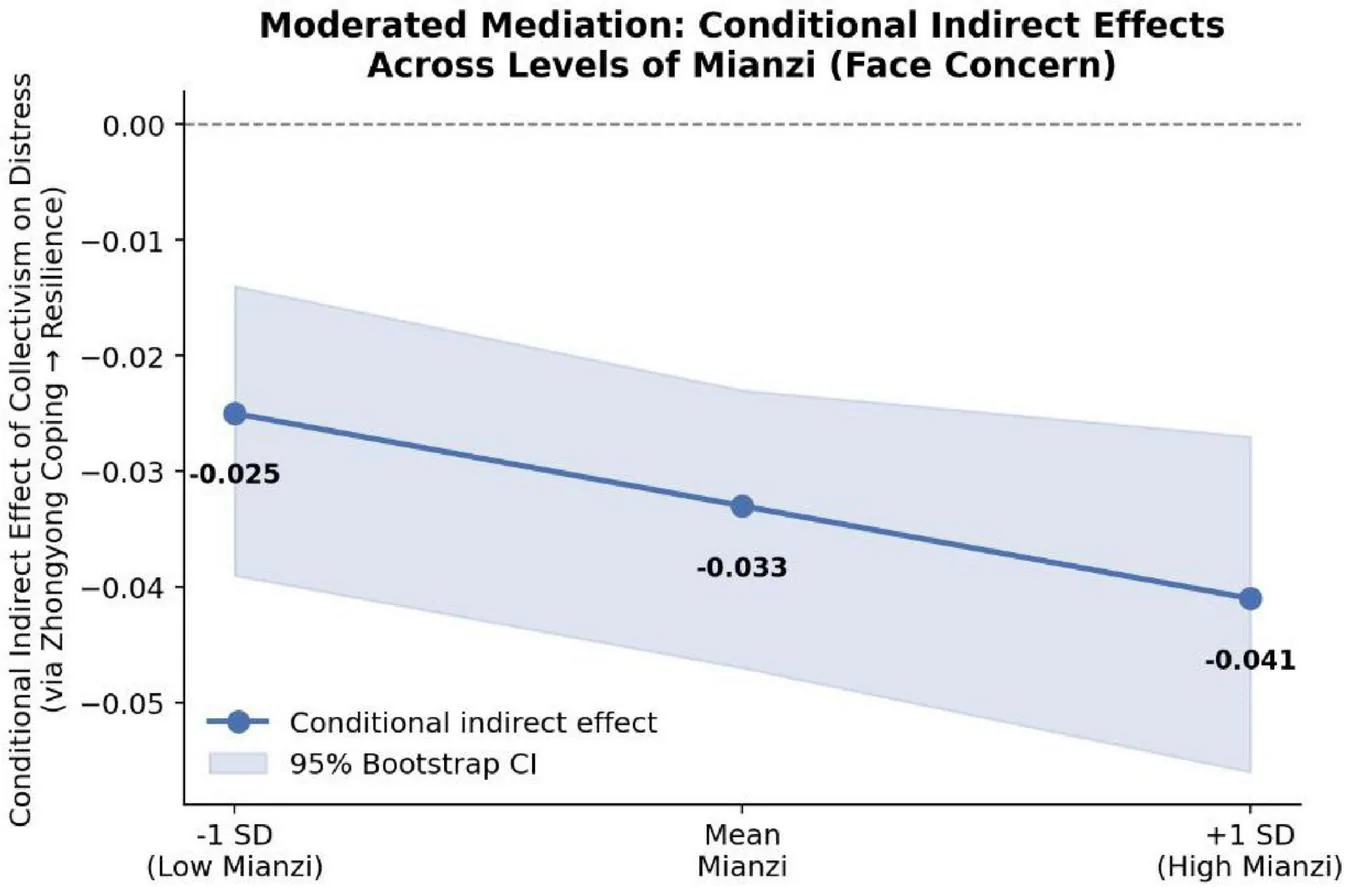
Conditional indirect effects. The y-axis represents the conditional indirect effect of collectivistic values on psychological distress via Zhongyong coping and resilience; the x-axis represents levels of Mianzi (face concern) at -1 SD, the mean, and +1 SD; shaded bands represent 95% bootstrap confidence intervals (see Table 1).

Moderation analysis further showed that face concern (Mianzi) significantly moderated the relationship between collectivistic values and Zhongyong coping. Table 11 showed that there were significant main effects of collectivistic values (β = 0.351, *p* < 0.001), Mianzi (β = 0.068, *p* = 0.041), and an interaction effect (β = 0.084, *p* = 0.010). The indirect effect of collectivistic values on the relationship between face concern and social norms was also found to be significantly positive, and gradually rising from negative 0.025 at low face concern to negative 0.041 at high face concern, as shown in Table 1. The significant index of moderated mediation (β = –0.008, 95% CI [–0.015, –0.002]) confirmed that teachers’ face concern fortified instead of detracted from the culturally based protective pathway, suggesting that teachers with higher levels of interpersonal harmony coping gained more psychological benefits from collectivistic values and coping with Zhongyong.

**TABLE 11:** Moderation of the cultural-values → coping path by Mianzi (face concern).

Path	B (std.)	SE	*t*	*p*
a1: Collectivism → Zhongyong coping (main effect)	0.351	0.033	10.50	<0.001
a3: Mianzi → Zhongyong coping (main effect)	0.068	0.033	2.05	0.041
a4: Collectivism × Mianzi (interaction)	0.084	0.033	2.58	0.010

Validation of the results through group comparisons and measurement validation further strengthened the results. Supplementary Table S3 showed no significant differences in psychological distress between municipal and school levels (*F* = 0.99, *p* = 0.372), but it was found that there were significant differences between the municipalities (*F* = 5.80, *p* = 0.003), with Xuzhou having the highest mean of distress.

Finally, Supplementary Table S4 presents the results of the hypothesis testing, which confirms support for all hypotheses (H1–H3). Overall, the results suggest that the occupational stressors contribute to psychological distress among teachers in China in different educational environments, while collectivist cultural values, Zhongyong coping, resilience and cultural motivations of facing have been interrelated protective factors that significantly protect against adverse psychological health outcomes in Chinese teachers in various educational contexts.

## 4 Discussion

The first goal of the present study was accomplished by showing that psychological distress has been a considerable mental health issue for teachers in Jiangsu Province. The prevalence estimates revealed that Moderate to Extremely Severe depression, anxiety, stress and psychological distress were common among participants, suggesting that mental health issues have not been present in isolated instances but have been a general issue of the workplace. This has been similar to the results of previous studies that found high levels of depression, anxiety, burnout and psychological distress among teachers in China and other educational settings (He et al., 2025; Fang et al., 2023). The same trend of increased psychological symptoms has been described in university educators and school staff around the world as well (Meeks et al., 2023; Hutchison et al., 2022). The educational implications of these results have been that teachers’ mental health needs must be considered a priority for institutions, as psychological wellbeing directly affects teaching efficacy, engagement in the teaching process and the quality of education.

The second goal was to explore the connection between occupational stressors, organizational support and psychological distress. Results showed that there was a significant positive correlation between workload and distress and a protective correlation between organizational support and distress, with work-family conflict also being a significant positive correlate of distress. Previous findings show that workload has been among the factors that most significantly predict teacher stress and lower psychological wellbeing (Zhang et al., 2023; Wang, 2024), which aligns with the results presented here. Similarly, the positive relationship between work-family conflict and distress aligns with earlier research that found work-family conflict has been related to emotional exhaustion and depression symptoms among Chinese teachers (Zhou and Li, 2022b; Zhong et al., 2025). The findings of the protective effect of organizational support also align with findings that found supportive school environments to be associated with higher work engagement, lower burnout, and higher job satisfaction (Chen et al., 2022; Liao et al., 2023). Therefore, workload management, equitable workload distribution, flexible working hours, and supportive leadership have been the priorities for teachers’ workload issues in the education sector.

Another major finding of this research was the confirmation of the role of collectivistic cultural values as significant psychological resources that help alleviate distress via adaptive coping and resilience. The mediation test showed that collectivistic values indirectly influenced psychological distress through the mediation of coping styles (Zhongyong coping) and mediated resilience. The results of this study add to the previous evidence that collectivist orientations have a positive effect on teachers’ wellbeing by creating a supportive psychological and organizational climate (Börü et al., 2025). The strong indirect pathways further support studies indicating that resilience plays a crucial role as a psychological process by which positive cognitive resources positively influence adaptive functioning (Liao and Hu, 2025). Additionally, the findings of the role of Zhongyong coping have been consistent with the evidence that Zhongyong thinking facilitates a process of adaptive cognitive regulation and positive emotional functioning that supports resilience (Zhou and Li, 2022a).

The study also fulfilled the goal of studying the culturally grounded psychological mechanism of Zhongyong coping between collectivistic values and resilience. Pathway from Zhongyong coping to resilience was significant, indicating that a coping style of balance and harmony allows teachers to recover better from the occupational adversity. The results support the studies that have shown that resilience has been fostered through the building up of psychological resources for effectively regulating stress (Liao and Hu, 2025). They are also congruent with evidence from previous studies showing that Zhongyong thinking improves psychological resilience and adaptive functioning in emotion regulation by cognitive reappraisal and positive affect (Zhou and Li, 2022a). In general, the findings highlight the need to include indigenous cultural coping strategies in teacher wellbeing interventions.

A further contribution of this study has been the fact that Mianzi has been found as a moderator in the conceptual model proposed in this study. This study found that face concern increased the positive link between collectivistic values and Zhongyong coping, which became increasingly protective against psychological distress. This indicates that interpersonal harmony concerns lead to an increase in culturally appropriate coping mechanisms. The discovery adds to the existing literature, indicating that collectivist contexts further reinforce the positive relationship between leadership and wellbeing with commitment of teachers (Bellibaş et al., 2024). Furthermore, this has been in line with research showing that culturally embedded psychological traits have been related to personal resources and have affected depression and adaptive functioning (Liu L. et al., 2024; Liu M. et al., 2024).

Important practical implications were also gained from the group comparison analyses. There was no significant difference in psychological distress between the different school levels; however, teacher psychological distress in Xuzhou was higher compared to that of Nanjing and Suzhou, indicating that the context and region have an impact on teacher wellbeing. Previous studies found that teachers in lower resource schools share similar geographical differences (Fang et al., 2023; He et al., 2025). Furthermore, the good measurement model reliability and validity have been in line with previous psychometric research on DASS-21 and other psychological scales in Chinese teachers (Cao et al., 2023). The results show that the integrated assessment system has been beneficial for the regular monitoring of teachers’ mental health in educational organizations.

Nevertheless, the following drawbacks have been noted: The cross-sectional design meant that there was no possibility of causal inferences despite the theoretical consistency of the proposed pathways. Longitudinal research would allow for the development of a clearer understanding of the temporal relationships between occupational stressors, cultural resources, coping, resilience, and psychological distress. Second, all variables were measured by self-report questionnaires, which has led to common method bias and socially desirable responding. Third, the results were obtained from participants in Jiangsu Province only, and thus cannot be generalized to other provinces in China, with different socio-economic and cultural differences. Lastly, even though culturally-specific constructs were combined in a single study and have not been studied together, some other contextual factors have contributed to differences in teachers’ wellbeing, such as leadership style, family support, psychological need satisfaction, and work engagement, that has been included in future studies to further understand the occupational mental health of Chinese teachers.

Several further limitations raised in review are also acknowledged. First, because all variables—including depression, anxiety, stress, and psychological distress—were assessed via self-report over a short recall window, the resulting rates reflect self-reported symptom levels rather than clinically confirmed diagnoses; the current design did not cross-validate these scores against clinician-administered or diagnostic interview measures, and future research should incorporate such validation. Second, given the sample’s gender composition (29.6% male, 70.4% female, broadly reflecting the gender distribution of the Chinese basic-education teaching workforce), gender was not modeled as a moderator of the study’s structural relationships; because collectivistic values, Zhongyong coping, and Mianzi may be enacted differently by men and women, future studies should test gender as a multi-group moderator of these pathways. Third, the group comparisons reported here focused on overall distress (DASS total); separate school-level and municipality-level comparisons for the depression, anxiety, and stress subscales were beyond the scope of the present analysis and are recommended for future work, alongside the municipality-level difference in depression prevalence already noted above. Fourth, marital status and marital transitions (e.g., divorce) were not measured in this survey; given that the sample’s mean age (37.6 years) falls within a period of the life course in which marital and family transitions are common in the Chinese context, future studies should collect marital-status data to test whether marital transitions help explain psychological distress among teachers alongside the occupational and cultural variables modeled here.

## 5 Conclusion

This study brings a holistic perspective of these occupational demands and culturally embedded psychological resources that have been found in teacher research on teacher psychological wellbeing, and adds to the current research on teacher mental health. This study adds to the existing occupational health literature, which has traditionally emphasized models centered on risk, by integrating the role of indigenous cultural constructs within an organizational stressor-mediating model and shows the importance of using culturally relevant constructs when studying mental health and wellbeing. The results highlight the need to implement a multi-dimensional intervention approach that focuses on workplace conditions, as well as on enhancing adaptive psychological skills and culturally congruent coping mechanisms. Practical implications for educational policymakers, school leaders and mental health practitioners in designing evidence-based wellbeing programs that “fit” Chinese educational contexts have also been drawn. Organizational reinforcements, preventive mental health programs, development of resilience and culturally sensitive professional training have been investments that have brought about healthier and more sustainable teaching environments. While the study has made contributions, it also calls for additional longitudinal, multi-regional, and mixed methods studies in the future to further test and validate the hypothesized relationships and examine other contextual factors. In conclusion, the study offers a solid basis for further research on culturally responsive teacher wellbeing and appropriate policies and interventions that have fostered psychological wellness in the education system.

## Data Availability

The original contributions presented in the study are included in the article/Supplementary material, further inquiries can be directed to the corresponding authors.
