# Supplementary material for: Prevalence of mental health issues among teachers in China: the role of cultural values and psychological resilience
Source: Front Psychol. 2026 Oct 1;17:1943721. doi: 10.3389/fpsyg.2026.1943721 (PMC13668755; doi:10.3389/fpsyg.2026.1943721)
Supplement: Supplementary file 1 [file Supplementary_file_1.docx]

**Supplementary Materials**

The following tables present secondary/descriptive analyses (discriminant-validity matrices, the group-comparison ANOVA, and the hypothesis-testing summary) that were moved out of the main text to improve readability, per reviewer recommendation. The core regression, mediation, and moderation models remain in the main text as Tables 1–10.

**Supplementary Table S1.** Discriminant Validity — Fornell-Larcker Criterion

|  | **Workload** | **WFC** | **Org. Support** | **Collectivism** | **Zhongyong** | **Mianzi** | **Resilience** | **Distress** |
| --- | --- | --- | --- | --- | --- | --- | --- | --- |
| **Workload** | **0.761** |  |  |  |  |  |  |  |
| **WFC** | 0.267 | **0.762** |  |  |  |  |  |  |
| **Org. Support** | −0.108 | −0.059 | **0.735** |  |  |  |  |  |
| **Collectivism** | 0.034 | 0.036 | −0.060 | **0.748** |  |  |  |  |
| **Zhongyong Coping** | −0.213 | −0.178 | 0.194 | 0.357 | **0.727** |  |  |  |
| **Mianzi (Face)** | 0.059 | 0.031 | −0.053 | 0.282 | 0.149 | **0.733** |  |  |
| **Resilience** | −0.245 | −0.214 | 0.258 | 0.173 | 0.469 | −0.010 | **0.696** |  |
| **Distress** | 0.407 | 0.378 | −0.297 | −0.143 | −0.410 | 0.045 | −0.486 | **0.624** |

**Supplementary Table S2.** Discriminant Validity — Heterotrait-Monotrait Ratio (HTMT)

|  | **Workload** | **WFC** | **Org. Support** | **Collectivism** | **Zhongyong** | **Mianzi** | **Resilience** | **Distress** |
| --- | --- | --- | --- | --- | --- | --- | --- | --- |
| **Workload** | — |  |  |  |  |  |  |  |
| **WFC** | 0.326 | — |  |  |  |  |  |  |
| **Org. Support** | 0.130 | 0.081 | — |  |  |  |  |  |
| **Collectivism** | 0.053 | 0.065 | 0.083 | — |  |  |  |  |
| **Zhongyong Coping** | 0.245 | 0.204 | 0.215 | 0.404 | — |  |  |  |
| **Mianzi (Face)** | 0.077 | 0.061 | 0.063 | 0.324 | 0.164 | — |  |  |
| **Resilience** | 0.277 | 0.242 | 0.282 | 0.193 | 0.498 | 0.056 | — |  |
| **Distress** | 0.469 | 0.434 | 0.330 | 0.163 | 0.444 | 0.068 | 0.518 | — |

**Supplementary Table S3.** Group Comparisons on Psychological Distress (DASS Total)

| **Comparison** | **F** | **p** | **η²** |
| --- | --- | --- | --- |
| Distress by School Level | 0.99 | .372 | 0.003 |
| Distress by Municipality | 5.80 | .003 | 0.015 |

**Supplementary Table S4.** Hypothesis Summary

| **Hypothesis** | **Key Statistic(s)** | **Decision** |
| --- | --- | --- |
| H1 | r = .41 (workload), .38 (work–family conflict), and −.30 (organizational support) with distress, all p < .001 (Table 6) | Supported |
| H2 | a1 (Collectivism→Coping) = 0.379; b1 (Coping→Resilience) = 0.369; b2 (Resilience→Distress) = −.257, all p < .001; sequential indirect effect = −.036, 95% CI [−.049, −.024] (Tables 7–8) | Supported (partial, not full, mediation) |
| H3 | Interaction a4 = 0.084, p = .010; index of moderated mediation = −.008, 95% CI [−.015, −.002] (Tables 9–10) | Supported — face concern strengthens (not weakens) the pathway |
